# Intermittent theta-burst stimulation combined with transcranial direct current stimulation once weekly for treatment-resistant depression: a case report

**DOI:** 10.1186/s13256-023-04152-0

**Published:** 2023-10-02

**Authors:** Pakorn Wivatvongvana, Chutimon Soonthornthum, Kittipong Kitisak

**Affiliations:** Department of Rehabilitation Medicine, Faculty of Medicine, Chiang Mai University, Chiang Mai, 50200 Thailand

**Keywords:** Treatment-resistant depression, iTBS, TMS, tDCS, PHQ-9

## Abstract

**Background:**

Single-time non-invasive brain stimulation was carried out using the two-technique approach on a patient suffering from treatment-resistant depression. Five treatment sessions given at weekly intervals resulted in a significant improvement in the Patient Health Questionnaire-9 score for up to 6 weeks. The findings of this study could pave the way for a more efficient less resource-intensive time- and budget-saving technique of employing non-invasive brain stimulation for patients with treatment-resistant depression by minimizing the number of stimulation sessions.

**Case presentation:**

A 67-year-old married non-Latino white American woman suffering from treatment-resistant depression received intermittent theta-burst stimulation in combination with transcranial direct current stimulation weekly for 5 consecutive weeks. Diagnostic transcranial magnetic stimulation showed an observable electrophysiological change. The patient reported a drastic improvement in Patient Health Questionnaire-9 score up until 6-week follow-up and expressed satisfaction with the treatment.

**Conclusions:**

This case study suggests that a streamlined protocol for using non-invasive brain stimulation could prove more effective for patients and healthcare providers in terms of safety in comparison to the present guidelines.

## Background

Transcranial magnetic stimulation (TMS) was first advocated for use in treating a major depressive disorder in 2008 [1]. The electromagnetic principle behind TMS shows that it can have a specific impact on cortical excitability. Since that time, it has expanded in its use to assist in a variety of neurorehabilitation scenarios. Neurostimulation occurs extensively via corticospinal neurons via fast-conducting direct waves and transsynaptic, later indirect, waves. The various components of corticofugal discharge have been used for diagnostic purposes in a variety of diseases of the central nervous system [2, 3].

The fundamental impact of transcranial direct current stimulation (tDCS) on neurons is a transient alteration in resting membrane potential toward depolarization or repolarization based on the changing streamwise direction related to axonal alignment. Anodal tDCS enhances the excitability of the underlying cortex, as evidenced by an increase in the amplitude of the motor-evoked potential (MEP) following transcranial magnetic stimulation, but cathodal tDCS reduces it. Past studies have shown that brain stimulation by tDCS is straightforward. Also, the effects of stimulation are not limited to the target site but are spread over adjacent areas [4, 5].

The standard technique of non-invasive brain stimulation (NIBS) for the treatment of treatment-resistant depression is either TMS via conventional high-frequency repetitive TMS (rTMS) [6, 7] or the newer, patterned intermittent theta-burst stimulation (iTBS) [8, 9] on consecutive weekdays for at least 4–6 weeks [6–9]. tDCS is intuitively used to deliver a small electrical current on 5 consecutive days over 2–3 weeks to have an antidepressive effect [5, 10, 11]. There have been few reports of the use of the combined techniques of NIBS. The number of treatment sessions is time-consuming, costly, and a burden in terms of health care. Integrating these two methods of NIBS to lessen the frequency of stimulation can be used for therapeutic effect, resulting in fewer patient visits and reduced health care resources.

## Case presentation

Mrs. C, a 67-year-old married non-Latino white American woman with a diagnosis of major depressive disorder (MDD) for more than 20 years and no full remission of the symptoms, was referred for care in March 2021. She suffered from low mood and energy, a lack of interest, trouble falling asleep, and difficulty concentrating on work or reading, which affected her daily life activities and social functioning. Her symptoms had shown no signs of improvement, despite her being adherent to antidepressants and anxiolytics (specifically bupropion 150 mg daily and alprazolam 1.5 mg at night). Her symptoms were easily aggravated by general life stressors. Thus, she was referred to our department for evaluation and treatment.

**Fig. 1:**
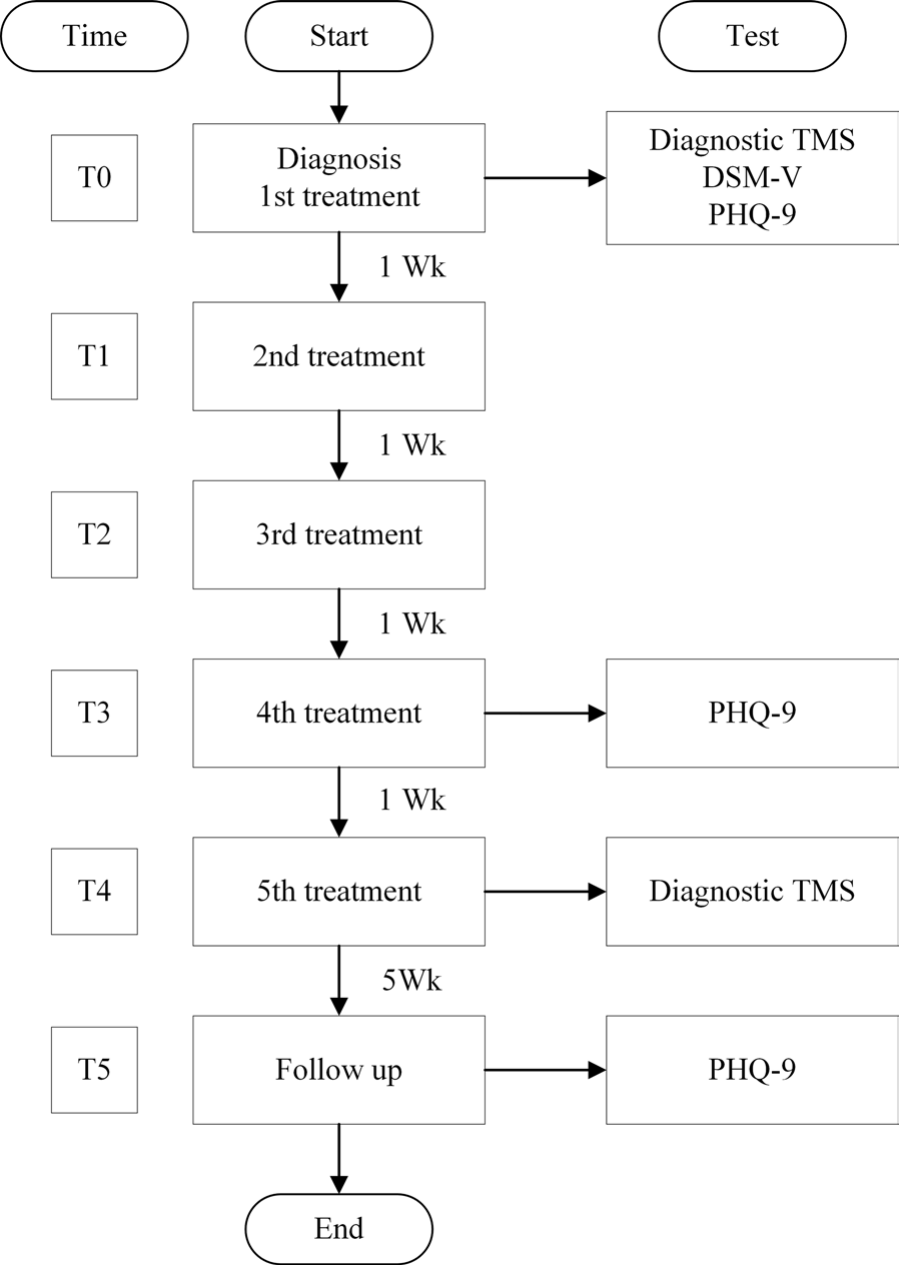
Study flow diagram

The diagnosis was assessed by the Diagnostic and Statistical Manual of Mental Disorders fifth edition (DSM-V) for MDD using a checklist to meet five out of nine criteria before the first treatment session on 4 March 2021 (T0). The patient presented with a sustained depressed mood, markedly diminished interest, significant weight loss, insomnia, fatigue, feelings of worthlessness, a diminished ability to think or concentrate, and recurrent thoughts of death. These symptoms generally cause significant distress in social areas of functioning and are not attributable to a substance or a medical condition, a psychotic disorder, or a manic or hypomanic episode [12]. The Patient Health Questionnaire-9 (PHQ-9) has been used as a reliable and valid tool for the diagnosis of a depressive disorder, and also during follow-up for measuring the severity of depression, as a self-administered patient health questionnaire [13]; see study flow in Fig. 1.

Before treatment, the diagnostic TMS demonstrated marked hypoexcitability of the left hemisphere due to a significant drop in motor-evoked potential [(MEP) 0.64 mV] and intracortical facilitation [(ICF) 1.43 mV] at 120% of motor threshold (MT), decreased short intracortical inhibition [(SICI) 1.02 mV], and a prolonged silent period (195 ms). Details of the normal diagnostic TMS parameters were described in our prior study [14]. Her PHQ-9 score was 23 out of 27, indicating severe depression.

The new iTBS treatment protocol consists of three-pulse TMS at 50 Hz in 20-ms intervals, which are repeated as a 2-second train at a frequency of 5 Hz every 10 seconds. The location is over the left dorsolateral prefrontal cortex (DLPFC) at 140% of MT, with a total of 2400 pulses for the first session. This is then followed by subsequent weekly iTBS at 100% of MT, a total of 1200 pulses for another four sessions. We immediately applied 20 minutes of tDCS with anodal stimulation over the left DLPFC and cathodal stimulation to the right supraorbital region after each iTBS session. The pharmacological regimen remained the same before and during the study process.

**Fig. 2:**
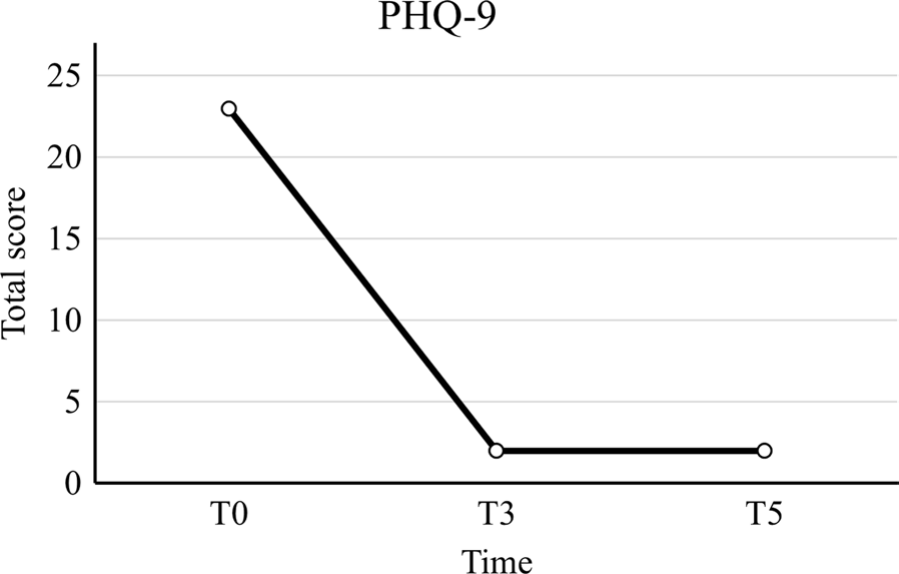
Effects of non-invasive brain stimulation on PHQ-9 score

The patient reported a 30% improvement in symptoms, mainly in mood and anxiety, after the first session (T0), which lasted for the following 6 days (T1). A 70% improvement was observed by the patient after the third (T2) and fourth (T3) sessions, corresponding with a significant decrease in the PHQ-9 score from 23 at the first session (T0) to 2 out of 27 in the fourth session (T3) and 2 out of 27 at the 6-week follow-up (T5), as shown in Fig. 2. Her mood, anxiety, concentration, relaxation, and ability to make decisions all improved, with the benefits enduring for at least 6 weeks after the treatment session ended. However, she still experienced some difficulty falling asleep. These symptoms were consistent with the follow-up diagnostic TMS results from the fifth session (T4). This demonstrated consistent MEPs, an increased SICI response (inhibition down to 0.29 mV), and a normal silent period (120 ms) of the left hemisphere.

The patient noted only pressure on the head in one session of the TMS treatment, which disappeared soon after stimulation. There was no sign of a headache. After removing the electrodes from the tDCS session, a burning sensation vanished. There was only minor redness on the scalp, which went away after a few hours.

## Discussion and conclusions

Conventional rTMS has been approved by the US Food and Drug Administration (US FDA) for treatment-resistant depression since 2008 [1], and iTBS has been approved since 2018 [15]. To date, rTMS has been reported as resulting in a remission rate of up to 40% with an odds ratio of 4.2 compared to sham [6, 7]. The new iTBS method is comparable to rTMS, the most powerful NIBS, but is a faster procedure [8, 9, 16]. Recently, accelerated TMS (aTMS) has been developed, which delivers five times the FDA-approved TMS dose for a full 5 consecutive days [17]. However, aTMS is beyond the scope of this study and needs more research to enable certification.

In contrast, tDCS statements were inconclusive, and that procedure has not been accepted by the US FDA. The number of studies to support the evidence is insignificant, and the results are controversial [5]. Systemic reviews reported a remission rate of up to 20% [10], with modest effect sizes and low efficiency for treating depression [11].

MDD has been associated with cortical hypoexcitability, which includes deficits in both glutaminergic and GABAergic pathways [18, 19]. This finding is consistent with the outcome of our diagnostic TMS, which reduced MEP, ICF, and SICI responses. However, we noticed a prolonged SP response, which might be a drug-induced effect, for example, from benzodiazepines, which are often used in patients with MDD [18, 20]. We decided to use iTBS as a newer and faster protocol in comparison to the prior high-frequency patterned rTMS [8, 9] to produce LTP-like synaptic plasticity [9] and enhance the hypoexcitability of the left hemisphere [9, 21, 22].

Even though the revised approach to employing iTBS remains unclear, we propose that the left DLPFC be used as the primary target for providing high-frequency rTMS, as indicated by a comprehensive analysis of the class I study and evidence level A from a prior systemic review [7]. The left DLPFC was carefully chosen as a preferential target for TMS to enhance the negative connectivity through a deeper dysfunctional neural network, that is, the subgenual anterior cingulate cortex, for an antidepressive effect [21–23].

For tDCS, we preferred to apply anodal tDCS over the left DLPFC and cathodal tDCS over the right supraorbital region, as is the usual protocol in the majority of studies [10, 11]. Even though the mechanism remains unclear, it appeared to increase the excitability of the hypofrontality state of the left hemisphere via the neuromodulation effect of tDCS [11, 24].

Contrary to Hebbian synaptic plasticity, we took precautions with the non-linear stimulus–response paradigm explained by the Bienenstock–Cooper–Munro (BCM) principle, which may transpose the resting threshold of one stimulus onto another when we repeat the two adjacent stimulations [25–27]. We truncate the pause duration between both stimulations as succinctly as possible to avoid a sliding threshold of postsynaptic neuronal conditioning as described in the protocol in our prior study [28].

From our results, which showed the PHQ-9 score declining from 23 to 2 out of 27 at the end of the treatment session and up to 6 weeks, the decrement is greater than that reported by the large observational study trial from 42 multi-cite US centers using conventional 10 Hz frequency rTMS in 307 patients. They found a decrease in PHQ-9 score from a mean score of 18.3 to 9.6 [29]. The clinical follow-up with a PHQ-9 score was congruent with the diagnostic TMS, which showed a more positive direction in hypoexcitability from the dominant hemisphere [14].

We postulate a new approach using these two techniques in this order. First and foremost, iTBS, as the most robust NIBS tool, primes the neuronal network at DLPFC, and is mainly focused on the specific area to produce the designated effect. Subsequently, anodal tDCS, as the less formidable NIBS instrument, but one that spreads the impact, works at the same spot to recruit neurons in a wider area of the neuronal network throughout. The aforementioned hypothesis was addressed in our prior study [28].

This technique appears to have a beneficially synergistic effect that sufficiently enhances neural plasticity throughout the week and is sustained for up to 6 weeks. The patient’s depressive symptoms were drastically improved by a significantly reduced number of stimulations from the standard protocol. However, it is too early to see whether the patient’s symptoms will still be in remission at 3–12 months or to report the maintenance session for NIBS treatment.

Adverse events detected in this study included pressure on the scalp during iTBS, which could be from the weight of the stimulation coil pressing on the patient’s head. A burning sensation, which is common during tDCS sessions, vanished after the stimulation was completed. Also, the erythema from the electrode faded within a few hours. These side effects were all transient. Critical adverse events such as seizures are rare and were not present in this study. This confirms the safety of the combined iTBS and anodal tDCS protocol for use with patients with treatment-resistant depression [4, 30–34].

In conclusion, we strongly believe that this new protocol of iTBS in combination with tDCS once weekly is safe, effective, less stressful, and more affordable for patients under limited resource health care systems. It warrants further research.

## Data Availability

The data that support the findings of this study are accessible from the Faculty of Medicine, Chiang Mai University, but access is limited since they were used under permission for the current study and are therefore not publicly available. However, data is available from the authors upon reasonable request and with permission from the Faculty of Medicine, Chiang Mai University.
