# Correction: Intermittent theta-burst stimulation combined with transcranial direct current stimulation once weekly for treatment‑resistant depression: a case report

**DOI:** 10.1186/s13256-024-04520-4

**Published:** 2024-04-05

**Authors:** Pakorn Wivatvongvana, Chutimon Soonthornthum, Kittipong Kitisak

**Affiliations:** Department of Rehabilitation Medicine, Faculty of Medicine, Chiang Mai University, Chiang Mai, 50200 Thailand

**Correction: Journal of Medical Case Reports (2023) 17: 415** 10.1186/s13256-023-04152-0

Following publication of the original article [1], an error was identified in the three places which incorrectly stated tetraburst instead of theta-burst.

The title currently reads:

Intermittent tetraburst stimulation combined with transcranial direct current stimulation once weekly for treatment‑resistant depression: a case report

The title should instead read:

Intermittent theta-burst stimulation combined with transcranial direct current stimulation once weekly for treatment‑resistant depression: a case report

The Abstract Case presentation currently reads:

**Case presentation**

A 67-year-old married non-Latino white American woman suffering from treatment-resistant depression received intermittent tetraburst stimulation in combination with transcranial direct current stimulation weekly for 5 consecutive weeks. Diagnostic transcranial magnetic stimulation showed an observable electrophysiological change. The patient reported a drastic improvement in Patient Health Questionnaire-9 score up until 6-week follow-up and expressed satisfaction with the treatment.

The Abstract Case presentation should instead read:

**Case presentation**

A 67-year-old married non-Latino white American woman suffering from treatment-resistant depression received intermittent theta-burst stimulation in combination with transcranial direct current stimulation weekly for 5 consecutive weeks. Diagnostic transcranial magnetic stimulation showed an observable electrophysiological change. The patient reported a drastic improvement in Patient Health Questionnaire-9 score up until 6-week follow-up and expressed satisfaction with the treatment.

The last paragraph of the Background section currently reads:

**Background**

The standard technique of non-invasive brain stimulation (NIBS) for the treatment of treatmentresistant depression is either TMS via conventional highfrequency repetitive TMS (rTMS) [6, 7] or the newer, patterned intermittent tetraburst stimulation (iTBS) [8, 9] on consecutive weekdays for at least 4–6 weeks [6–9]. tDCS is intuitively used to deliver a small electrical current on 5 consecutive days over 2–3 weeks to have an antidepressive effect [5, 10, 11]. There have been few reports of the use of the combined techniques of NIBS. The number of treatment sessions is time-consuming, costly, and a burden in terms of health care. Integrating these two methods of NIBS to lessen the frequency of stimulation can be used for therapeutic effect, resulting in fewer patient visits and reduced health care resources.

The last paragraph of the Background section should instead read:

**Background**

The standard technique of non-invasive brain stimulation (NIBS) for the treatment of treatment resistant depression is either TMS via conventional highfrequency repetitive TMS (rTMS) [6, 7] or the newer, patterned intermittent theta-burst stimulation (iTBS) [8, 9] on consecutive weekdays for at least 4–6 weeks [6–9]. tDCS is intuitively used to deliver a small electrical current on 5 consecutive days over 2–3 weeks to have an antidepressive effect [5, 10, 11]. There have been few reports of the use of the combined techniques of NIBS. The number of treatment sessions is time-consuming, costly, and a burden in terms of health care. Integrating these two methods of NIBS to lessen the frequency of stimulation can be used for therapeutic effect, resulting in fewer patient visits and reduced health care resources.

The three occasions have been updated above and the original article [1] has been corrected.

## References

[1] Wivatvongvana P, Soonthornthum C, Kitisak K (2023). Intermittent theta-burst stimulation combined with transcranial direct current stimulation once weekly for treatment-resistant depression: a case report. J Med Case Reports.

[5] Lefaucheur J-P, Antal A, Ayache SS, Benninger DH, Brunelin J, Cogiamanian F (2017). Evidence-based guidelines on the therapeutic use of transcranial direct current stimulation (tDCS). Clin Neurophysiol.

[6] Perera T, George MS, Grammer G, Janicak PG, Pascual-Leone A, Wirecki TS (2016). The clinical TMS society consensus review and treatment recommendations for TMS therapy for major depressive disorder. Brain Stimul.

[7] Lefaucheur J-P, Aleman A, Baeken C, Benninger DH, Brunelin J, Di Lazzaro V (2020). Evidence-based guidelines on the therapeutic use of repetitive transcranial magnetic stimulation (rTMS): an update (2014–2018). Clin Neurophysiol.

[8] Blumberger DM, Vila-Rodriguez F, Thorpe KE, Feffer K, Noda Y, Giacobbe P (2018). Effectiveness of theta burst versus high-frequency repetitive transcranial magnetic stimulation in patients with depression (THREE-D): a randomised non-inferiority trial. Lancet.

[9] Chung SW, Hoy KE, Fitzgerald PB (2015). Theta-burst stimulation: a new form of TMS treatment for depression?. Depress Anxiety.

[10] Berlim MT, Van den Eynde F, Daskalakis ZJ (2013). Clinical utility of transcranial direct current stimulation (tDCS) for treating major depression: a systematic review and meta-analysis of randomized, double-blind and sham-controlled trials. J Psychiatr Res.

[11] Palm U, Hasan A, Strube W, Padberg F (2016). tDCS for the treatment of depression: a comprehensive review. Eur Arch Psychiatry Clin Neurosci.

